# Effects of technological running shoes versus barefoot running on the intrinsic foot muscles, ankle mobility, and dynamic control: a novel cross-sectional research

**DOI:** 10.1016/j.bjpt.2024.101092

**Published:** 2024-06-16

**Authors:** María García-Arrabé, Iván Batuecas-Sánchez, Silvia de Vidania, María Bravo-Aguilar, Beatriz Ruiz-Ruiz, Carlos Romero-Morales

**Affiliations:** Faculty of Sport Sciences, Universidad Europea de Madrid, Villaviciosa de Odón, Madrid, Spain

**Keywords:** Footwear, Kinematics, Ultrasound

## Abstract

**Background:**

Technological running shoes have become increasingly popular, leading to improvements in performance. However, their long-term effects on foot musculature and joint mobility have not been thoroughly studied.

**Objective:**

To compare the activation of the intrinsic foot muscles between runners wearing technological footwear and barefoot runners. Secondary objectives included assessing ankle dorsiflexion (DF) range of motion (ROM) and dynamic postural control in both groups.

**Methods:**

A cross-sectional study was conducted involving 22 technological footwear runners and 22 barefoot runners. Ultrasonography was used to measure the thickness of the plantar fascia (PF) and the quadratus plantae (QP), abductor digiti minimus (ADM), abductor hallucis (AH), and flexor hallucis longus (FHL) muscles. Ankle mobility and dynamic postural control were also recorded.

**Results:**

Ultrasonography measurements showed statistically significant differences for PF thickness (mean difference [MD]: -0.10 cm; 95% CI: -0.13, -0.05 cm), QP cross-sectional area (CSA) (MD: -0.45 cm^2^; 95% CI: -0.77, -0.12 cm^2^), ADM CSA (MD: -0.49 cm^2^; 95% CI: -0.70, -0.17 cm^2^), and FHL thickness (MD: 0.82 cm; 95% CI: 0.53, 1.09 cm), with all measurements being lower in the group wearing technological footwear compared to the barefoot runners. Ankle DF ROM was also significantly greater for the barefoot runners (MD: -5.1°; 95% CI: -8.6, -1.7°).

**Conclusions:**

These findings suggest potential implications for the foot musculature and ankle mobility in runners using technological footwear.

## Highlights

• Tech footwear linked to reduced foot muscle vs. barefoot.
• Barefoot group shows significantly higher ankle dorsiflexion ROM.
• No significant dynamic balance differences found between groups.

## Introduction

Over the past few years, a notable trend has been observed towards the integration of advanced technologies in the field of sports footwear, aimed at enhancing athletes' performance. Currently, the most innovative and widely used technologies are the implementation of advanced foam midsoles, carbon-fibre plates, responsive soles, and heel cups. These technologies are designed to provide motion control and stability, enhance the shoe's elastic properties, deliver superior cushioning, and optimize energy return.^1^

The development of innovative technology in shoes has led to significant improvement in performance, with many athletes setting both personal and world records in long-distance competitions.^2^ For instance, Kelvin Kiptum achieved the world record time of 2h35sec at the 2023 Chicago Marathon while wearing advanced technology shoes.^3^ Furthermore, data from the Strava application indicate that runners who utilize models with high technology such as the Vaporfly 4% or Next% can decrease their marathon and half-marathon times by 4% to 5% and have as much as a 73% to 75% probability of surpassing their personal best compared to when using conventional running shoes.^4^ However, to date, no studies have been conducted to examine the medium and long-term effects of using this type of technological footwear on the intrinsic muscles of the foot, as well as on ankle mobility and stability.

In contrast, the barefoot running movement emerged a few decades ago, aiming to prevent injuries and promote a more natural running style that emphasizes the development of intrinsic foot muscles as a key factor in foot and ankle stability and control.^5^ Running barefoot or using minimalist footwear has been associated with improvements in proprioceptive motor regulation^6^ and better alignment of the lower limbs.^7^^,^^8^ Although several researchers have identified significant changes in terms of biomechanics,^9^ kinetics,^10^ and muscle activation^11^^,^^12^ when transitioning to minimalist footwear or running barefoot, the time elapsed thus far appears to be insufficient to verify long-term changes in muscle architecture and foot mobility.^13^

Ultrasound imaging (USI) has been widely used to assess the architecture (size, shape, thickness, and cross-sectional area [CSA] of anatomical structures.^14^^,^^15^ Decreased thickness and CSA of the abductor hallucis brevis (AHB) and flexor hallucis brevis (FHB) have been reported in individuals with hallux valgus.^16^ Romero et al. also reported that the thickness of the AHB and flexor digitorum brevis (FDB), as well as the CSA of the FDB and FHB, were greater in individuals diagnosed with Achilles tendinopathy when compared to the healthy group.^17^ In addition, the plantar fascia (PF) had greater thickness and CSA in patients with flat fleet.^18^ Currently, several authors support the use of USI as a non-invasive, relatively non-expensive, safe, and a valid portable tool to evaluate soft tissues and musculoskeletal conditions.^19^^,^^20^

The main objective of this study is to compare the PF and intrinsic foot muscles, such as the quadratus plantae (QP), abductor digiti minimus (ADM), AHB, and FHL assessed by USI, as well as ankle mobility and dynamic postural control in experienced runners using technological footwear versus experienced barefoot runners. We hypothesize that participants who run with technological footwear exhibit reduced thickness and CSA of the foot's intrinsic musculature, along with decreased ankle range of motion (ROM) and decreased dynamic motion control.

## Methods

### Study design

This cross-sectional observational study was performed following the Strengthening the Reporting of Observational Studies in Epidemiology (STROBE) guidelines for reporting observational studies^21^ between March 2022 and May 2023 at the University Europea of Madrid and Los Molinos Physiotherapy Center.

### Participants

A sample of 44 runners was recruited (22 who wore technological footwear and 22 who ran barefoot). The inclusion criteria comprised participants being 18 to 55 years of age and who had been running regularly for at least 1 year before the evaluation. Exclusion criteria were self-reported or medical record of injuries in the lower limb in the previous 6 months.

Participants were allocated to groups as follows:

*Technological Footwear Group*: Runners employing advanced athletic footwear, characterized by features such as motion control, stability, and performance-enhancing devices (e.g., carbon plates),^2^ were assigned to this group. These shoes are notable for their ability to provide technological control and support during running.

*Barefoot Group:* This group included runners who engaged in completely barefoot running or utilized minimalist footwear with an index of 90%^22^ or higher, such as huaraches or "FiveFingers" type shoes. These types of footwear provide a running experience almost similar to running barefoot.

### Sample size calculation

To determine the sample size, a pilot study with 20 participants was conducted consisting of two groups using the QP muscle thickness as outcome variable. The mean (SD) thickness for the 10 participants for the technological footwear group was 1.86 (0.41) cm compared to 2.3 (0.58) cm for the 10 participants for the barefoot group. Subsequently, the GPower software was utilized, setting a confidence level of 95%, a statistical power of 0.80, an effect size of 0.87, and an α error of 0.05. Using these parameters, the required sample size was determined to be 22 participants per group.

### Ethical statement

Ethical approval was obtained from the Ethics Committee University Europea of Madrid (CIPI/22.182). All participants included in the study signed the informed consent form. The study was conducted in accordance with the Declaration of Helsinki for human experimentation.

### Ultrasound imaging

A high-quality ultrasound system (LOGIQ V2; GE Healthcare, United Kingdom) fitted with an 8 to 13 MHz range linear transducer (12L-RS, 33 mm footprint) was used to perform the USI assessment of the intrinsic foot muscles. The scanning was conducted on the dominant lower limb and three measurements were taken at each site, removing the probe between each measurement.

The ultrasonography assessment of the foot muscles was based on the guidelines by Mickle et al..^23^ First, participants were in the prone position with the feet overhanging the end of the plinth. PF thickness was measured by placing the transducer on the long axis between the calcaneus and the second toe, and the images were acquired at the thickest point. With the probe in this position, the thickest part of the QP muscle was located, often proximal to the spring ligament, and a still image was taken to measure the thickness. For the evaluation of the CSA of the QP muscle the transducer was rotated 90°. To evaluate the ADM muscle thickness the probe was placed at the insertion of the muscle towards the tuberosity of the 5th metatarsal and, for the CSA the probe was rotated 90° in the same position. For the AH muscle thickness evaluation, participants were placed lying in supine position and the transducer was located at the insertion point of the muscle, directed towards 1–2 cm proximal to the navicular tuberosity, and for the CSA the probe was rotated 90° at the same location. To explore the FHL muscle, participants were lying in a supine position with the knee flexed and hip in external rotation. For the thickness evaluation, the probe was placed in the middle of the tibia, perpendicular to the long axis and moved posteriorly to find the thickest part of the muscle.

### Active ankle mobility

Ankle dorsiflexion (DF) ROM was tested by myROM mobile app.^24^ From a half-kneeling position, with the dominant limb on the ground and hands on their waist, participants were asked to lean forward as far as possible. The phone was placed on the tibia and the angle of tibial inclination (indirect measure of ankle dorsiflexion) was retrieved on the screen. Three measurements were taken for each participant, with 30 s between consecutive measurements.

### Dynamic postural control

The Y-Balance Test (YBT) was used to assess dynamic postural control.^25^ Three pieces of tape were oriented in a Y-shape fashion: the first piece of tape was oriented anteriorly to measure dynamic stability for the anterior direction. The other 2 pieces were angled 135° from the first one and were used to measure dynamic stability in the posteromedial and posterolateral directions. Participants were asked to stand barefoot on the dominant limb with hands on their waist, and reach with the swing limb in the anterior, posteromedial, and posterolateral direction while maintaining balance on the support limb. Three repetitions were performed, with a 30-second rest between each, and the mean of the three trials in each direction was recorded.

### Statistical analysis

The statistical analysis was developed by the SPSS package v.22.0 (IBM, Armonk, NY: IBM Corp). First, Shapiro-Wilk test was used to assess normality of data distribution. Second, descriptive analyses were done for all participants together and then separately for the two groups. Finally, a comparative analysis between the technological footwear and barefoot groups was developed. Mean, standard deviation (SD) with the Student‘s t-test for independent samples and median, interquartile range (IR) with Mann-Whitney U test were performed for parametric and non-parametric data, respectively. Levene´s test was employed to assess the equality of variances. An α error of 0.05 (95% CI) and desired power of 80% (β error of 0.2) were used throughout the study.

## Results

Sociodemographic data did not show statistically significant differences between groups (Table 1). Ultrasonography measurements of the intrinsic foot muscles (Table 2) showed statistically significant differences for PF thickness (mean difference [MD]: −0.10 cm; 95% CI: −0.13, −0.05), QP CSA (MD: −0.45 cm^2^; 95% CI: −0.77, −0.12), ADM CSA (MD: −0.49 cm^2;^ 95% CI: −0.70, −0.17), and FHL thickness (MD: 0.82 cm; 95% CI: 0.53, 1.09), with all values being lower in the footwear technology group versus the barefoot running group. Ankle DF ROM was significantly greater for the barefoot running group (MD: −5.12; (95% CI: −8.6, −1.7). Dynamic balance values between groups did not differ: YBT-A (MD: 4.10 cm; 95% CI: −0.85, 9.06), YBT-L (MD: 1.19 cm; 95% CI: −4.44, 6.82), and YBT-M (MD: −0.69 cm; 95% CI: −6.06, 4.69) (Table 3).

**Table 1:** Sociodemographic data and weekly running distance.

	Technological footwear (*n* = 22)	Barefoot (*n* = 22)	Mean difference (95% CI)
Age, y	39.36 (9.26)	41.38 (8.65)	−2.02 (−7.32, 3.28)
Weight, kg	73.00 (10.39)	69.71 (10.63)	3.29 (−2.92, 9.50)
Height, m	1.76 (0.54)	1.73 (0.79)	0.03 (−0.37, 0.43)
BMI, kg/m^2^	23.37 (2.42)	23.01 (2.30)	0.36 (−1.04, 1.76)
Running distance, km/week	38.8 (7.40)	37.6 (9.60)	1.20 (−3.87, 6.27)

Results are mean (SD) and mean difference (95 % CI).

**Table 2:** Ultrasound imaging measurements.

Measurement	Technological footwear (*n* = 22)	Barefoot (*n* = 22)	Mean difference (95% CI)
PF thickness (cm)	0.26 (0.05)	0.41 (0.05)	−0.10 (−0.13, −0.05)
QP CSA (cm^2^)	1.52 (0.39)	2.11 (0.68)	−0.45 (−0.77, −0.12)
QP thickness (cm)	0.78 (0.19)	0.76 [0.39]†	0.16 (0.00, 0.32)
AH CSA (cm^2^)	2.01 (0.74)	2.39 (0.54)	−0.13 (−0.52, 0.25)
AH thickness (cm)	0.89 (0.28)	0.97 [0.99] †	0.20 (−0.12, −0.44)
ADM CSA (cm^2^)	1.08 [0.50] †	1.53 (0.36)	−0.49 (−0.70, −0.17)
ADM thickness (cm)	0.65 (0.13)	0.73 [0.16]†	−0.05 (−0.16, −0.05)
FHL CSA (cm^2^)	1.31 (0.40)	1.37 (0.23)	0.22 (−0.67, −1.11)
FHL thickness (cm)	1.16 (0.40)	0.16 [0.55]†	0.82 (0.53, 1.09)

Abbreviations: ADM, abductor digiti minimi; AH, abductor hallucis; CSA, cross sectional area; FHL, flexor hallucis longus; QP, quadratus plantae.

Results are mean (SD) and mean difference (95 % CI) as used.

† Results are median [IQ].

**Table 3:** Range of motion and balance measurements.

Measurement	Technological footwear (*n* = 22)	Barefoot (*n* = 22)	Mean differences (95 % CI)
Ankle DF ROM (°)	38.84 (6.15)	43.96 (5.19)	−5.12 (−8.58, −1.65)
YBT-A (cm)	89.01 (8.88)	84.90 (7.33)	4.10 (−0.85, 9.06)
YBT-L (cm)	78.47 (9.15)	77.28 (9.35)	1.19 (−4.44, 6.82)
YBT-M (cm)	74.16 (8.49)	74.84 (9.17)	−0.69 (−6.06, 4.69)

Abbreviations: DF, dorsiflexion; ROM, range of motion; YBT, Y-balance test.

Results are mean (SD) and mean difference (95 % CI) as used.

## Discussion

This study examined the muscular architecture and ankle mobility between technological footwear and barefoot runners. The results revealed significant differences for the thickness of the PF and FHL; the CSA of the QP and ADM; and for ankle DF ROM, with all values being greater in barefoot runners. No significant differences in dynamic balance were found. Our results support the notion that the use of technologically advanced footwear vs barefoot may lead to reduced intrinsic musculature development^26^ and support the hypothesis that barefoot running promotes greater dorsal flexion of the ankle and long-term strengthening of the intrinsic foot musculature.^27^^,^^28^

Current research supports that an increase in muscle thickness and CSA is associated with improved strength. Therefore, the results found in the present study suggest that barefoot running for at least two years can promote the strengthening of the intrinsic musculature of the foot, as demonstrated by the greater muscle thickness compared to the group using technological footwear.

These findings are consistent with previous studies that have observed positive changes in the intrinsic foot musculature with the minimalist footwear.^29^^,^^30^ With the use of the Vibram FiveFinger Bikila during walking over the course of 24 weeks, a significant increase in AH muscle thickness was observed^,^^31^ which plays an essential role in foot stabilization and the prevention of running-related injuries.^32^

The influence of footwear, the strengthening of intrinsic foot musculature, and the development of the arch have been studied in the past decades.^33^ The type of footwear can impact the stiffness of the longitudinal arch and intrinsic muscle strength of the foot.^34^ In addition, significant changes in CSA and ABH and ADM volume were observed in runners who transitioned to minimalist footwear within 12 weeks.

Furthermore, Taddei^35^ has emphasized that strengthening intrinsic muscles can have an impact on running mechanics and enhance overall running performance.^36^ The authors found significant correlations between muscle volume and anteroposterior propulsive force.

Technological footwear is primarily engineered to provide cushioning, arch support, and stability. For instance, cushioning incorporates specialized midsole materials to absorb shock and distribute pressure. Arch support maintains the foot's natural arch shape and improves stability, contributing to the overall biomechanics of the foot.^37^ These design features aim to mitigate impact forces, enhance comfort, and reduce the risk of injury.^38^^,^^39^ However, this additional protection can have negative effects on the musculature, as it reduces the need for these muscles to naturally activate and strengthen,^40^ potentially compromising foot stability and responsiveness during running.^41^

Barefoot runners showed significantly greater ankle DF ROM as compared to those using advanced footwear technology. An increase of ankle joint flexibility may be related to a need for impact absorption. This finding agrees with previous research which suggest that running barefoot could promote greater mobility in the foot and ankle joints due to a sensory stimulation and freedom of movement provided by the absence of restrictive footwear.^42^

The results of this study revealed an average decrease of 4.85° in ankle ROM in runners using technological footwear as compared to barefoot runners. It is possible that the restriction of movement in this group is influenced by a combination of factors. Firstly, technological footwear often has a high heel-to-toe drop, which implies a significant height difference between the heel and forefoot.^43^^,^^44^ This design may promote a more forward-leaning posture, which in turn limits ankle DF and shortens the posterior chain musculature.^45^ Furthermore, some models of technological footwear incorporate restrictive technologies, such as structural reinforcements in the back of the shoe, which can restrict natural ankle movement.^46^

Increasing ankle DF can help runners maintain optimal subtalar joint position by decreasing the degree of subtalar joint pronation and its consequences, which could increase the risk of injury. In addition, individuals with limited ankle DF experience varying degrees of altered kinematics and dynamics at the pelvis, hip, knee and foot during walking and jogging. Limited ankle DF alters the movement pattern of the lower extremity during walking and jogging, decreasing the body's ability to propel itself forward, which can increase the risk of injury.^47^, ^48^, ^49^

The results of our study suggest that barefoot running may be an effective intervention to have a wide DF and thus may help reduce the occurrence of certain dysfunctions of the lower limb. Sorrentino et al.^50^ have investigated the mobility strategy in the modern human talus revealing that the morphology of this bone varies according to differences in locomotor and cultural behavior. They concluded that the morphological variation of the talus is related to the use of constrictive footwear in post-industrial society, which reduces the ROM of the ankle. This stands in contrast to hunter-gatherers, where the talus shape displays a more flexible profile, likely attributable to the habit of regularly walking barefoot, even across uneven terrain.^50^

In a systematic review with meta-analysis, Almeida et al.^51^ have explored biomechanical disparities in foot impact patterns during running, particularly comparing natural rearfoot and forefoot strikes. They observed that rearfoot strikers typically make initial ground contact with a dorsiflexed foot, while forefoot strikers land with a plantarflexed foot. These findings indicate varying ankle mobility requirements based on individual biomechanics and foot strike patterns.

Regarding dynamic postural control, no significant differences were found between groups for the YBT. This suggests that the use of advanced footwear technology does not significantly influence balance compared to barefoot running. The YBT primarily assesses dynamic stability requiring contribution from all hip, knee, ankle, and foot. In addition, it is important to note that balance and stability depends on the complex interaction of various sensory, neuromuscular, and cognitive systems in the human body, not solely on strength.^52^^,^^53^ Perhaps the complexity of the interaction of all these systems involved in the Y-balance test could explain the lack of differences found in adjusting posture and maintaining stability during the test in both studied groups. Another factor to be considered is that balance and stability are also influenced by movement patterns developed throughout our lives.^54^ Human body was designed based on specific motor patterns in response to the demands and stimuli to which is exposed. Therefore, there may have been considerable variability within each group in terms of balance and stability skills, which could have diluted any potential effects of the footwear used.

### Limitations and future lines

The cross-sectional design of this study limits the ability to establish causal relationships between the variables examined. A longitudinal study would be necessary to observe changes over time regarding footwear and participants' evolution.

Additionally, the absence of a non-runner control group and the lack of data on gait patterns and ankle/foot mechanics during running are significant limitations. These gaps hinder understanding of how different footwear conditions affect running biomechanics.

Future research could benefit from incorporating ultrasound elastography and electromyography studies focused on intrinsic foot muscles, correlating them with running biomechanics.

### Clinical relevance

Our results suggest that technological footwear may limit the development of intrinsic foot musculature and ankle DF mobility, while barefoot running appears to promote the development of stronger intrinsic musculature and greater ankle ROM. However, it is important to note that longitudinal studies are necessary to adequately assess the effects of both footwear and barefoot conditions over time.

## Conclusions

The results of the present study reported that running with technological shoes is associated with a decrease in PF and FHL thickness, as well as QP and ADM CSA, and ankle ROM compared to barefoot running. These results highlight the significant impact of footwear choice on various foot parameters.

## Conflicts of interest

The author declares no conflicts of interest.
